# Biological characteristics of stem cells derived from burned skin—a comparative study with umbilical cord stem cells

**DOI:** 10.1186/s13287-021-02140-z

**Published:** 2021-02-17

**Authors:** Reinhard Dolp, Gertraud Eylert, Christopher Auger, Ayesha Aijaz, Yufei Andy Chen, Saeid Amini-Nik, Alexandra Parousis, Andrea-Kaye Datu, Marc G. Jeschke

**Affiliations:** 1 Sunnybrook Research Institute, Toronto, Canada; 2 Department of Psychiatry, Queen’s University, Kingston, Canada; 3 Institute of Medical Science, University of Toronto, Ontario, Canada; 4 Division of Plastic, Aesthetic and Reconstructive Surgery, Medical University of Graz, Graz, Austria; 5 Department of Laboratory Medicine and Pathobiology (LMP), University of Toronto, Toronto, Canada; 6 SGS Harrison Research Laboratories, SGS North America, New York Metropolitan Area, Union, NJ USA; 7 Department of Immunology, Ross Tilley Burn Centre, Sunnybrook Health Sciences Centre, 2075 Bayview Ave., Toronto, ON M4N 3M5 Canada; 8 Division of Plastic and Reconstructive Surgery, Department of Surgery, Faculty of Medicine, University of Toronto, Toronto, Canada; 9 Ross Tilley Burn Centre, Sunnybrook Health Science Centre, Toronto, Canada

**Keywords:** Mesenchymal stromal/stem cells, • Burn-derived mesenchymal stromal/stem cells, • Umbilical cord mesenchymal stromal/stem cells, Cell Therapy, Skin regeneration, Wound healing, Burn(s), Cell biologic function

## Abstract

**Introduction:**

Burned human skin, which is routinely excised and discarded, contains viable mesenchymal stromal/stem cells (burn-derived mesenchymal stromal/stem cells; BD-MSCs). These cells show promising potential to enable and aid wound regeneration. However, little is known about their cell characteristics and biological function.

**Objectives:**

This study had two aims: first, to assess critical and cellular characteristics of BD-MSCs and, second, to compare those results with multipotent well-characterized MSCs from Wharton’s jelly of human umbilical cords (umbilical cord mesenchymal stromal/stem cells, UC-MSCs).

**Methods:**

BD- and UC-MSCs were compared using immunophenotyping, multi-lineage differentiation, seahorse analysis for glycolytic and mitochondrial function, immune surface markers, and cell secretion profile assays.

**Results:**

When compared to UC-MSCs, BD-MSCs demonstrated a lower mesenchymal differentiation capacity and altered inflammatory cytokine secretomes at baseline and after stimulation with lipopolysaccharides. No significant differences were found in population doubling time, colony formation, cell proliferation cell cycle, production of reactive oxygen species, glycolytic and mitochondrial function, and in the expression of major histocompatibility complex I and II and toll-like receptor (TLR).

**Importance, translation:**

This study reveals valuable insights about MSCs obtained from burned skin and show comparable cellular characteristics with UC-MSCs, highlighting their potentials in cell therapy and skin regeneration.

**Supplementary Information:**

The online version contains supplementary material available at 10.1186/s13287-021-02140-z.

## Introduction

The skin is the largest organ of the human body and has many essential functions, such as regulating systemic metabolism and providing a protective barrier against external insults. The loss of skin following a burn is the main determinant for life or death as it leads to a high risk of multi-organ failure, infection/sepsis, and hypermetabolism [1]. Therefore, the timely and adequate surgical removal of burned skin as a source of infection and inflammation, and subsequent wound coverage to induce regeneration are imperative for survival.

Despite all advances in regenerative medicine and tissue engineering, an ideal replacement for the damaged skin, especially after large burn injuries, has yet to be discovered. Mesenchymal stromal/stem cells (MSCs) [1] are a promising source for cell-based therapies [2]. They possess an ability of self-renewal and cell differentiation, and several other unique characteristics such as secretion of paracrine factors that promote angiogenesis, reepithelialization, granulation tissue formation, and modulate inflammation [3]. They were found to regenerate the epidermis [4] and dermis significantly [5], have anti-inflammatory and anti-fibrotic/scarring [6] potential, and are able to resemble skin pigmentation [7] and renew skin appendages [8].

Amongst the most commonly used MSC [9–11] sources are the bone marrow [12], the adipose tissue [13], and the perinatal tissue [14], including the umbilical cord (UC) [15–18]. Previously, umbilical cord-derived mesenchymal stromal/stem cells (UC-MSCs) were described as having remarkable healing effects when injected into non-healing burn (scarred) wounds [19]. UC-MSCs are associated with superior benefits compared to other adult MSC sources such as their immunosuppressive properties [20], their multipotency [21], and their ability to accelerate scarless healing [22, 23].

Recent discoveries indicated that burned tissues can provide a source of viable MSCs [6, 24–26]. Adipose-derived stem cells (ASCs) from burn fat [6, 27–30] seem to be unaffected by the thermal injury and are usable for further culturing [27]. MSCs extracted from full-thickness dermal skin [6, 28] of patients (burn-derived MSCs, BD-MSCs) showed promising healing results in small-sampled murine and porcine trials without signs of immunologic rejection [6]. These BD-MSCs have the potential to be a completely autologous source for skin regeneration without ethical concerns or the need for further harvesting methods while being readily available in every burn patient. However, the exact biological characteristics as well as the essential factors contributing to the healing potential for thermal damage from these cells have yet to be explored. This study aims to determine the critical biological characteristics of BD-MSCs and compared to cells from the very multipotent source in regenerative medicine, human UC-MSCs [6].

## Materials and methods

### Burned skin and umbilical cord tissue, cell isolation

Discarded burned skin (without subcutaneous fat, surgical dermatome) was received from full-thickness burned patients after receiving written consent from the operation room from the Ross Tilley Burn Centre of the Sunnybrook Hospital Toronto, Canada. The tissue was washed in PBS with 1% Ab/Am. Afterward the burned skin (necrosis, “upper/loose layer”) was scratched off (easily with a scalpel) and the dermis (“rigid layer”) was minced with a scalpel into small pieces, and transferred into a 50-ml Falcon containing human collagenase 1 (Worthington Biochemical Corporation, USA), dispase II (Life Technologies Corporation, USA), trypsin (Life Technologies Corporation, USA), DMEM medium with 1% Ab/Am and was incubated in a rotator at 37.5 °C for 60 min. Then the cell-enzyme mix was diluted with PBS 50:50 and filtered through a 100-μm cell strainer (Falcon® 100 μm Cell Strainer, Corning, USA). The filtered cell-enzyme mix was centrifuged at 1000 rpm for 10 min, supernatant was discarded, cells were plated in T-75 flasks with 8 ml of DMEM medium enriched with 1% Ab/Am and 10% fetal bovine serum (FBS, Gibco™ fetal bovine serum, Life Technologies Corporation, USA). The medium was changed every 2–3 days, upon reaching 80% confluency for further passaging and experiments (Supplementary Material, Figure 1).

Umbilical cords were received after written consent and donation from the Obstetrical and Gynecology Department from Sunnybrook Hospital, which were stored in Dulbecco’s modified Eagle’s medium (Gibco™ DMEM, Thermo Fischer Scientific, Canada) enriched with 1% antibiotic-antimycotic solution (Gibco® Antibiotic-Antimycotic, Thermo Fisher Scientific, Canada) for a maximum period of 24 h in the fridge at 4 °C before processing. The umbilical cords were washed with PBS containing 1% Ab/Am. Small pieces of avascular tissue (< 5 mm) were extracted with a dermal scalpel from Wharton’s jelly from the umbilical cord stroma, as previously described from our stem cell laboratory [31, 32]. The tissue pieces were placed in a 6-well plate and incubated for 7–10 days, until outgrow was visible. The medium was changed partially after 4–5 days, and afterward fully every 2–3 days with Dulbecco’s modified Eagle’s medium (Gibco™ DMEM) supplemented with 10% FBS, 1% l-Glutamine, and 1% Ab/Am, upon seeing a colony outgrow for further passaging and experiments.

For the following experiments, cells from three different burn patients (BD cells) and three different umbilical cords (UC cells) were assessed, after initial cell extraction and cell sorting based on MSC surface markers from passages 1 and 3–4, respectively, in triplicates.

### Flow cytometry assay

Cell sorting with flow cytometry was performed using cell surface markers for MSCs according to the International Society for Cellular Therapy [15]; live cells using DAPI were selected and gated with the negative markers CD34−/CD11b−/CD45− (FITC) (Invitrogen), CD19−/HLA-DR− (AF700, PE-Cy7) (eBioscience), and positive markers were gated for CD73+ (PE) (eBioscience), CD90+ (BV510) (eBioscience) and CD105+ (APC) (eBioscience) using a BD LSR II Flow Cytometer with the BD FACSDIVA™ SOFTWARE (BD Biosciences, Canada) as previously shown (Cheng & Eylert et al., 2020) [33].

### MSC differentiation assay

Cells were seeded with a passage number 1 with 6000 cells per 24-well plates.

#### Adipogenic differentiation

Cells were cultured in low-glucose DMEM supplemented with 10% FBS, 1% Ab/ Am, 1 mM of 3-isobutyl-1-methylxanthine, 10 μg/mL of insulin, 60 μM of indomethacin, and 1 μM of dexamethasone. Cells were placed in an incubator at 37 °C in 5% CO_2_ for 10 days. The medium was changed three times weekly. Staining was performed, and cultured cells were fixed with 4% paraformaldehyde and stained with Oil Red O (Sigma-Aldrich, Canada).

#### Chondrogenic differentiation

Cells were cultured in low-glucose DMEM supplemented with 10% FBS, 1% Ab/Am, 1 mM of sodium pyruvate, 0.1 mM of ascorbic acid-2-phosphate, 1% insulin-transferrin- selenium, 100 nM of dexamethasone, and 10 ng/mL of TGF-β3. Cells were placed in an incubator at 37 °C in 5% CO_2_ for 10 days. The medium was changed twice weekly. Staining was performed, and cultured cells were fixed with 4% paraformaldehyde and stained with Alcian Blue (Santa Cruz Biotechnology, Canada).

#### Osteogenic differentiation

Cells were cultured in low-glucose DMEM supplemented with 10% FBS, 1% Ab/Am, 0.05 mM ascorbic acid-2-phosphate, 10 mM β-glycerophosphate, and 100 nM dexamethasone. Cells were placed in an incubator at 37 °C in 5% CO_2_ for 10 days. The medium was changed twice weekly. Staining was performed, and cultured cells were fixed with 4% paraformaldehyde and stained with Alizarin Red S (Sigma-Aldrich, Canada).

#### Measurement was performed

Adipogenic differentiation potential [(measured as red-oil-o positive cells × 100/total amount of cells per visual field)], chondrogenic differentiation potential [(measured as Alcian Blue-positive area / area visual field)], osteogenic differentiation [(measured as Alizarin Red-positive area / area visual field)].

### Population doubling time

Cells were seeded into 24-well culture plates (100 cells per plate) and assessed. Adhesive cells were counted after 24 (Ni) and 48 h (Nn). Population doubling time (PDT) was calculated with the following formula: PDT = 48 h/((logNn) − (logNi)/log2). For cells with the passage number of 1, the attached cells were counted at 24 h and 48 h and the PDT was calculated using the same formula.

### Colony-forming unit (CFU) assay

Cells were seeded in 6-well plates in duplicates at three different cell concentrations (100, 500, and 1000 cells/100 mm^2^), measurements were made counting per unit area (cells/area in mm^2^) of the entire well. The number of colonies larger than 3 mm in diameter was manually counted. Cells were cultured for 2 weeks with one time change of growth medium and 10% FBS. Staining was performed using 0.5% crystal violet (Thermo Fisher Scientific, Canada) in methanol for 15 min at room temperature and washed twice with PBS, followed by imaging.

### Proliferation via bromodeoxyuridine (BrdU) staining

Cells were cultured in 8 chamber slides (Falcon™ Chambered Cell Culture Slides, Fisher Scientific, Canada) until reaching confluency of 80–90% before staining. Each biological sample was cultured and stained in doublets. BrdU (Cell Signaling Technology Inc., Canada) was added to the culture medium (1:200) and incubated with the cells for 12 h prior. The cultured cells were fixed in 4% paraformaldehyde (Electron Microscopy Sciences, USA) followed by permeabilization with 0.25% Triton X-100 (BioShop Canada Inc., Canada) and incubated in 1.5 M hydrochloric acid (Sigma Aldrich, Canada). To prevent unspecific binding, PBS was added with 1% bovine serum albumin (WISENT Inc., Canada). The samples were incubated with the primary antibody (BrdU (Bu20a) Mouse mAb #5292, Cell Signaling Technology Inc., Canada) at 4 °C overnight, followed by a 1-h long incubation at room temperature in the secondary antibody (Alexa Fluor® 488 dye, Thermo Fischer Scientific, Canada). Afterward, the slides were mounted with mounting medium containing DAPI (VECTASHIELD Antifade Mounting Medium with DAPI, Vector Laboratories, USA).

### Cell cycle analysis

Cells with confluency of 90% were used and analyzed with a Propidium Iodide Flow Cytometry Kit for Cell Cycle Analysis (ab139418, Abcam, Canada) strictly according to manufacturer’s instructions. Cells were trypsinized, fixed in 75% ethanol, and incubated with propidium iodide and RNase for 30 min. DNA staining was analyzed via flow cytometry, with the BD™ LSR II flow cytometer (BD Biosciences, Canada) using the BD FACSDIVA™ SOFTWARE (BD Biosciences, Canada).

### Reactive oxygen species (ROS) expression

Cells were stained for 2′,7′-dichlorofluorescin diacetate (DCFDA) and cultured in 96-well plates (Corning® 96 Well Flat Clear Bottom Black Polystyrene TC-Treated Microplates, Corning Incorporated, USA) until reaching confluency of 95%, and assessed in triplicate. Cells were incubated with 25 μM 2′,7′-dichlorofluorescin diacetate (DCFDA) in PBS for 45 min at 37 °C. As a positive control, cells were additionally exposed to 0.1 mM H_2_O_2_ (Laboratories Atlas Inc., Canada) for 30 min. Fluorescence intensity was measured with a plate reader (Synergy™ H4 Hybrid Multi-Mode Microplate Reader, BioTek Instruments Inc., USA).

### Cell viability and apoptosis

Cell viability was determined by a Live/Dead Viability/Cytotoxicity Kit (Thermo Fisher Scientific, Canada). Extracted cells from burned tissue and from umbilical cord at passage 1 were seeded in a 96-well with a cell density of 100,000 cells per well in triplicates and were cultured with 150 μl of DMEM medium enriched with 1% Ab/Am and 10% FBS for 48 h before staining and imaging. Live cells were stained with Calcein-AM (green channel) and dead cells with EThD1 (red channel) according to the manufacturer’s protocol and were visualized with Zeiss, Z1 spinning disc confocal microscope.

Apoptosis was assessed using TdT-mediated dUTP Nick-End Labeling (Tunel) staining. Cells were cultured in 8 chamber slides until reaching confluency of 80–90% before staining. The DeadEnd™ Fluorometric TUNEL System-Kit (Promega Corporation, USA) was used to detect fragmentation of DNA. The cultured cells were fixed in 4% paraformaldehyde, followed by permeabilization with 0.25% Triton X-100. After pH equilibration with the equilibration buffer, cells were incubated for 60 min in the dark at 37 °C in the TdT reaction mix containing recombinant deoxynucleotidyl transferase (rTdT), fluorescein-12-dUTP, and equilibration buffer. The positive control was incubated with DNAse I before incubating in the TdT reaction mix. After staining, the cell-containing slides were mounted with DAPI-medium.

### Glycolytic and mitochondrial function

The glycolytic and mitochondrial function was assessed with the Seahorse XF96 analyzer (Seahorse Bioscience, USA) using the Seahorse XF Glycolysis Stress Test Kit (Seahorse Bioscience Inc., USA) and the Seahorse XF Cell Mito Stress Test Kit (Seahorse Bioscience, USA). Both kits required additional XF96 cell culture plates, sensor cartridges, and XF base medium from the same company. Cells at a passage number of 3–4 from three different umbilical cords and three different burn patients were seeded in the XF96 cell culture plates (30,000 cells/well) and incubated for 12 h in standard cell culture medium (DMEM, 10% FBS, 1% Ab/Am) at 37 °C. Each biological sample was analyzed in six replicates.

#### Mitochondrial function

The standard medium was washed off and replaced by XF base medium supplemented with 25 mM glucose, 2 mM glutamine, and 1 mM sodium pyruvate. Oligomycin, carbonyl cyanide 4-(trifluoromethoxy) phenylhydrazone (FCCP), rotenone, and antimycin A were loaded in the recommended dosages into the sensor cartridge. Sensor cartridge and cell-containing culture plate were inserted into the Seahorse XFe96. Oxygen consumption rate (OCR) along with extracellular acidification rate (ECAR) was measured at baseline and after sequential addition of oligomycin, FCCP, and rotenone and antimycin A.

#### Glycolytic function

Standard medium was washed out and replaced by XF base medium supplemented 2 mM glutamine. Glucose, oligomycin, and 2-deoxy-d-glucose (2-DG) were loaded in the recommended dosages into the sensor cartridge. Simultaneously to the mitochondrial stress kit, oxygen consumption rate (OCR) along with extracellular acidification rate (ECAR) was measured at baseline and after the injection of the pre-loaded substances.

#### Data analysis

The measured data were analyzed using the supplied XF mito stress test report generator and the XF glycolysis stress test report generator.

### Immunogenicity and immunoreactivity

For evaluation of major histocompatibility complexes (MHC) I and II and toll-like receptor (TLR) 4, cells were used, after fixing with 0.01% paraformaldehyde and incubation for 1 h at − 4 °C with the conjugated antibodies TLR-4 (eBioscience, Thermo Fisher Scientific Inc., Canada), MHC II (eBioscience, Thermo Fisher Scientific Inc., Canada), and MHC I (eBioscience, Thermo Fisher Scientific Inc., Canada) together with flow buffer consisting of HBSS with 1% bovine serum albumin. After washing, cells were analyzed via flow cytometry.

For further assessment, qPCR for toll-like receptor (TLR) 1–10 was performed using Primers as demonstrated (Table 1). Additionally, peripheral blood mononuclear cells from two different patients were used as a positive control. Total RNA was isolated using TRIzol reagent (Invitrogen, Canada) according to the manufacturer’s instructions. RNA concentration and quality were assessed using spectrophotometry (Nanodrop 2000, Thermo Fischer Scientific Inc., Canada). RNA with a 260/280 ratio > 1.8 was accepted. mRNA expression was quantified using StepONE Plus PCR System (Applied Biosystems, California, USA) and Bio-Rad Advanced Universal SYBR® Green Supermix (Bio-Rad, CA, USA) according to the manufacturer’s directions. First-strand cDNA synthesis from 2 μg of total RNA was performed with random primers using High-Capacity cDNA Reverse Transcription Kits (Applied Biosystems, USA) according to the manufacturer’s instructions. Forward and reverse primers were optimized to verify primer efficiency, and dissociation melt curves were analyzed for primer specificity. All samples were run in duplicate, simultaneously with negative controls that contained no cDNA. Primers were ordered from Life Technologies Inc., Canada. See Table 1 for the full sequence of the individual primers. Optimization was performed to determine a 1:20 dilution ratio of plasma in nuclease-free sterile water, and 2 μl of starting material was used per reaction. All samples were run in duplicate, simultaneously with negative controls. Transcript levels were normalized to GAPDH and analyzed using the 2^−ΔΔCt^ method. Statistical significance was calculated on Δct values. We chose GAPDH as a housekeeping gene, since it is one of the most widely used reference genes in high-impact studies and considered a classical housekeeping gene [34].

**Table 1:** Primers for toll-like receptor 1–10

Target	Forward primer	Reverse primer
TLR 1	GCACCCCTACAAAAGGAATCTG	GGCAAAATGGAAGATGCTAGTCA
TLR 2	CTGGTAGTTGTGGGTTGAAGCA	GATTGGAGGATTCTTCCTTGGA
TLR 3	TTAAAGAGTTTTCTCCAGGGTGTTTT	AATGCTTGTGTTTGCTAATTCCAA
TLR 4	CCCCTTCTCAACCAAGAACC	ATTGTCTGGATTTCACACCTGGAT
TLR 5	TGCTAGGACAACGAGGATCATG	GAGGTTGCAGAAACGATAAAAGG
TLR 6	AGGCCCTGCCCATCTGTAA	GCAATTGGCAGCAAATCTAATTT
TLR 7	GCTATTGGGCCCATCTCAAG	TCCACATTGGAAACACCATTTTT
TLR 8	TCAGTGTTAGGGAACATCAGCAA	AACATGTTTTCCTTTTTAGTCTCCTTTC
TLR 9	GGGAGCTACTAGGCTGGTATAAAAATC	GCTACAGGGAAGGATGCTTCAC
TLR 10	TTTACTCTGGGACGACCTTTTCC	ATAAGCCTTACCACCAAAAGTCACA

### Secretion profile

For the detection of cytokines, chemokines, growth factors, and immunomodulatory proteins, the HCYTOMAG-60 K MILLIPLEX MAP Human Cytokine/Chemokine Magnetic Bead Panel - Immunology Multiplex Assay (EMD Millipore Corporation, Germany) was used. This kit enables the detection of sCD40L, EGF, FGF-2, Flt-3 ligand, Fractalkine, G-CSF, GM-CSF, GRO, IFN-α2, IFN-γ, IL-1α, IL-1β, IL-1ra, IL-2, IL-3, IL-4, IL-5, IL-6, IL-7, IL-8, IL-9, IL-10, IL-12 (p40), IL-12 (p70), IL-13, IL-15, IL-17A, IP-10, MCP-1, MCP-3, MDC (CCL22), MIP-1α, MIP-1β, PDGF-AB/BB, RANTES, TGF-α, TNF-α, TNF-β, VEGF, Eotaxin/CCL11, and PDGF-AA. The basic principle of this kit is that the antigens of interest are bound to color-coded magnetic beads on the one side, and a fluorescent conjugate (Biotin-Streptavidin) on the other side. The color of the attached fluorescent bead is specific for the antigen of interest; the emitted fluorescence from the Biotin-Streptavidin system is directly proportionate to the amount of the bound antigen of interest. Cells were cultured in 6-well plates until they reached a confluency of 90–95%, then they were incubated for 48 h in the fresh standard medium. A second group was incubated for 48 h in standard medium containing 1 μg/ml lipopolysaccharide (LPS; lipopolysaccharide from *Pseudomonas aeruginosa* 10, Sigma Aldrich, Canada). Experimental design adhered to the Immunology Multiplex Assay protocol. The medium of the different groups was filled in a 96-well plate and mixed with the magnetic beads and the fluorescent conjugate. All components that were not bound to the beads were washed off, and the fluorescent conjugate was analyzed using the Luminex 100® Milliplex® Analyzer (EMD Millipore Corporation, Germany). We excluded secreted proteins if all cells of one biological group were not in the detectable range of the machine.

### Statistical analysis and graphical representation

Statistical analysis was done with Microsoft Excel 2016 and Prism GraphPad Version 5.0a for Mac. Two groups were compared with an unpaired *t*-test, more than two groups with a one-way ANOVA with a post hoc Tukey test. A *p* value of *p* < 0.05 was considered statistically significant. All graphs are made with Prism GraphPad Version 5.0a for Mac and display mean ± SEM. The analysis for flow cytometry was done in FlowJo™ v10 for MAC and Prism 8 for Mac OS X.

## Results

### Mesenchymal stromal/stem cells

**Fig. 1:**
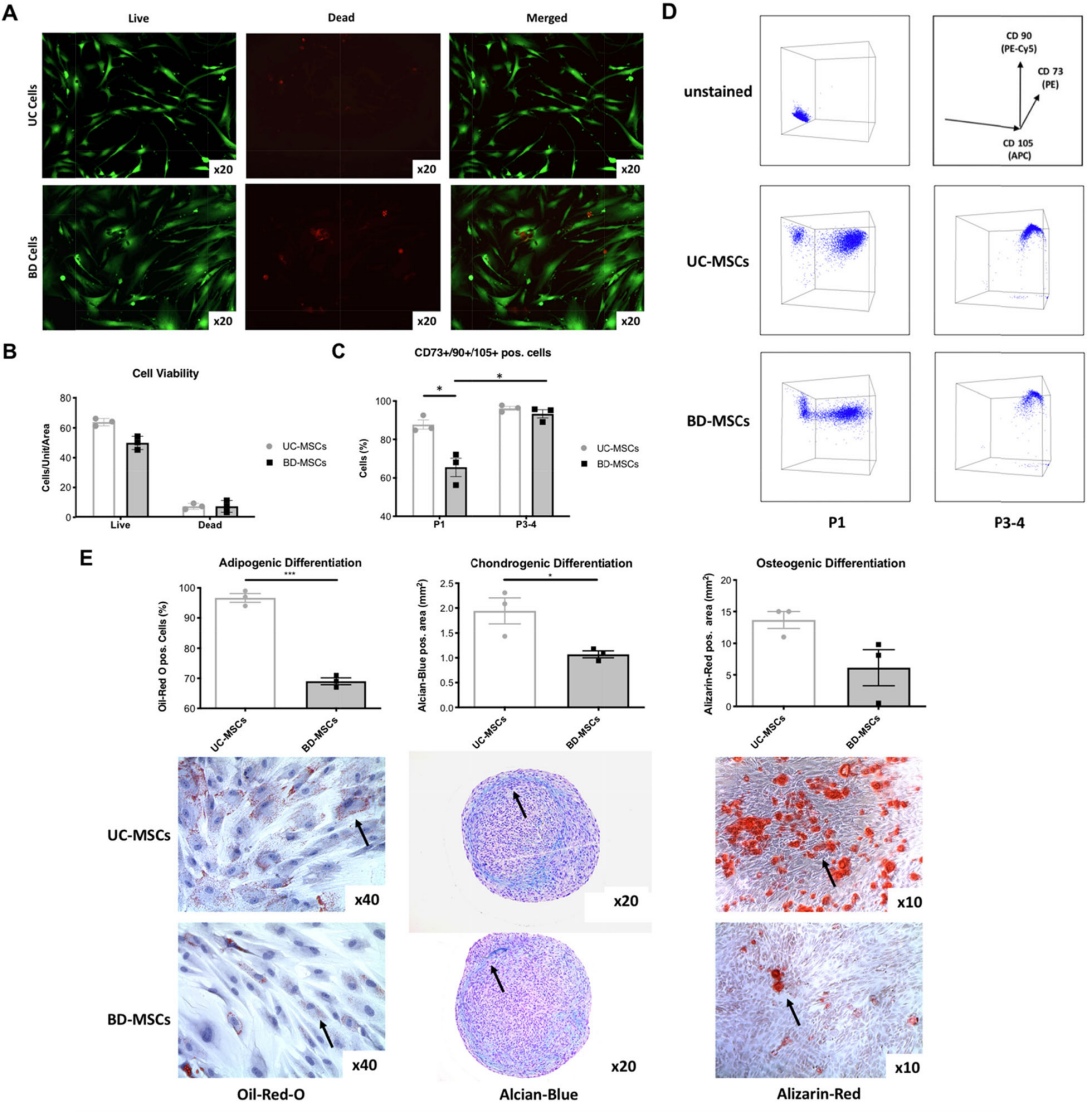
Mesenchymal stromal/stem cells. **a** Cell viability, 48 h after seeding, Passage 1. **b** Quantification of the cell viability, live/dead assay. **c** Immunophenotyping of the positive MSCs surface markers, passage 1 and 3–4. **d** Flow cytometry for positive MSC surface markers, note that negative markers were gaited out. **e** Statistical presentation of adipogenic, chondrogenic, and osteogenic differentiation. Images were taken after 10 days of differentiation into adipogenic, chondrogenic, and osteogenic lineage. Statistical significance is indicated with asterisks: *p value ≤ 0.05, ** *p* ≤ 0.01, and *** *p* ≤ 0.001. No asterisks represent *p* > 0.05. *N* = 3 for each group (=cells from 3 different patients per group); triplicates per biological sample. Graph: Mean with SEM

Cells from full-thickness burned human dermal skin (BD-MSCs) and Wharton’s jelly of human stroma of the umbilical cords (UC-MSCs) were extracted and cultured. The enzymatic method (60 min) for BD-MSCs yielded: 16,140 ± 5418 attached cells per square centimeter of processed burned skin after 24 h vs. the conventional method (7–10 days) for UC-MSCs from Wharton’s jelly cell yielded: 125,000 ± 20,600 attached cells per square centimeter of umbilical cord measured via trypan blue. Cell viability was assessed 48 h after seeding the same amount of cells at passage 1. Quantification was assessed; the viability varied between 91 and 95%. There appeared to be no statistical significant difference between the UC- and BD-MSC groups (Fig. 1a, b).

Characterization of MSCs was done with flow cytometry to assess the surface marker profile defined by the International Society for Cellular Therapy [35] as previously shown [33], as well as differentiation potential into osteogenic, chondrogenic, and adipogenic tissues. Both cell groups displayed the surface marker profile of MSCs, at different times of cultivation (Fig. 1d, e); pos. expression of Cluster of Differentiation (CD) 73/90/105, neg. expression CD11b/CD34/CD45/CD19/Human Leukocyte Antigen – DR isotype (HLA-DR).

At passage 1, 66 ± 5% (SEM %) of BD-MSCs displayed this surface marker profile compared to 89 ± 5% of UC-MSCs (*p* = 0.025). At passage 3 to 4, > 90% of cells in both cell groups displayed the surface marker profile for MSCs: 93 ± 2% (SEM %) in BD-MSCs and 96 ± 2% (SEM %) in UC-MSCs (*p* = 0.015) (Fig. 1c). Cells from both groups were able to differentiate into the mesenchymal lineages such as chondrogenic, adipogenic, and osteogenic (Fig. 1e). In comparison, BD-MSCs showed an overall lower differentiation potential compared to UC-MSCs; statistically significant for adipogenic (*p* = 0.001) and chondrogenic differentiation (*p* = 0.03).

### Cell function

Basic biological cell function was assessed via population doubling time (PDT), colony-forming behavior, cell proliferation, cell cycle phases, secretion of reactive oxygen species (ROS), and apoptosis.

**Fig. 2:**
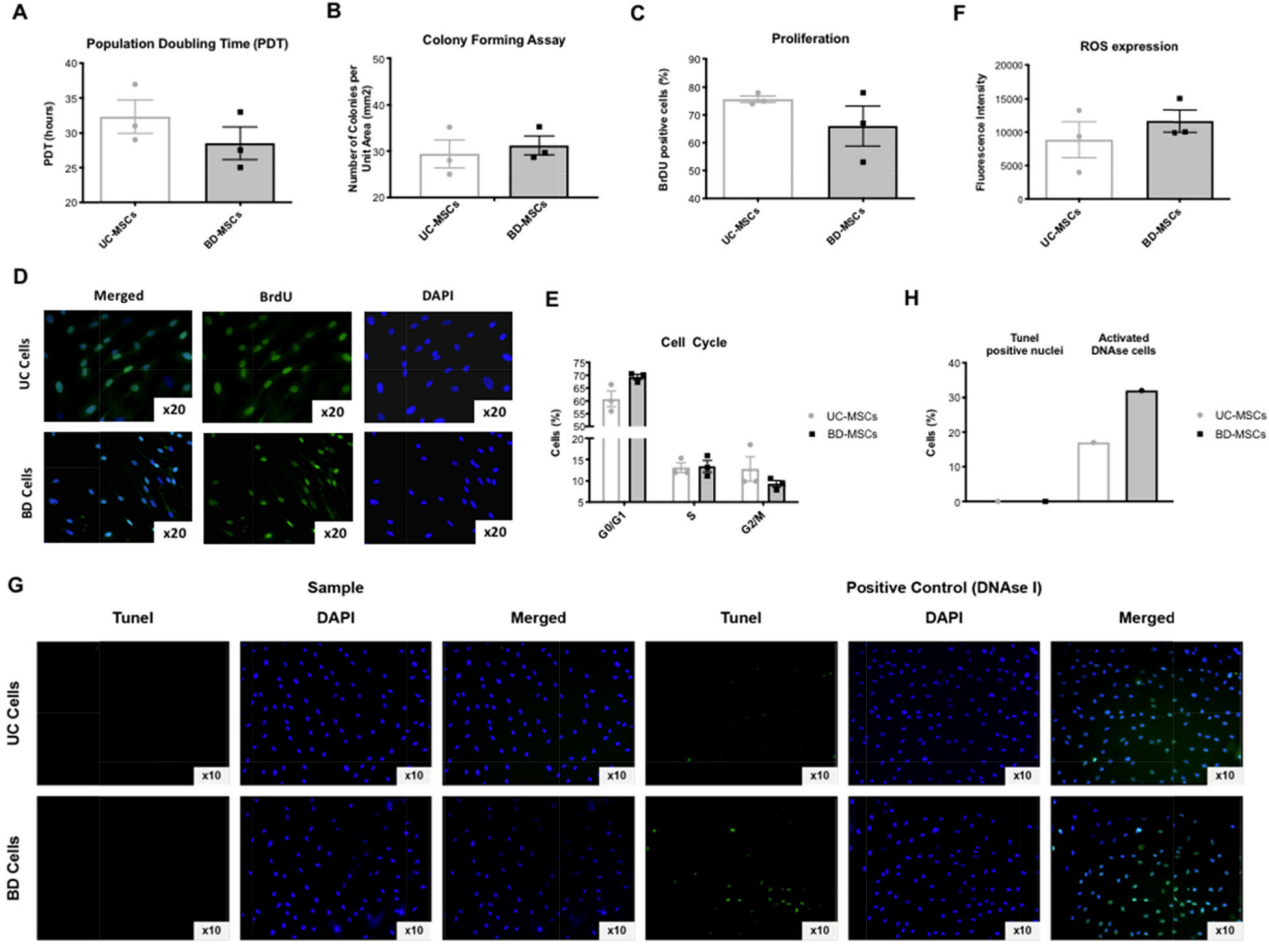
Cell function. **a** Population doubling time (PDT). **b** Colony-forming Assay. **c**, **d** Proliferation assessed with BrdU staining and Images merged, BrdU, and DAPI. **e** Cell cycle distribution. **f** Reactive oxygen species (ROS) expression. **g**, **h** Apoptosis assessed with TUNEL staining of the sample and the positive control (DNAse I). Statistical significance is indicated with asterisks: * *p* value ≤ 0.05, ** *p* ≤ 0.01, and *** *p* ≤ 0.001. No asterisks represent *p* > 0.05. *N* = 3 for each group (=cells from 3 different patients per group); triplicates per biological sample. Graph: Mean with SEM

There was no statistical difference between BD-MSCs and UC-MSCs in terms of PDT (28 ± 4 h and 32 ± 3 h, respectively) (Fig. 2a), number of colonies formed after 20 days (31 ± 4 colonies and 29 ± 5 colonies) (Fig. 2b), cell proliferation 12 h after plating (67 ± 13% and 75 ± 2% BrdU positive cells) (Fig. 2c, d), distribution within the different cell cycle phases (G0/1 phase: 69 ± 2% and 61 ± 5%, S phase: 13.4 ± 2% and 13.1 ± 2%, G2/M phase: 9.3 ± 1% and 12.8 ± 5%) (Fig. 2e), and production of ROS (11,685 ± 2904 fluorescence intensity and 8871 ± 4675) (Fig. 2f). None of the cultured cells showed apoptosis in the TUNEL staining (Fig. 2g, h).

### Mitochondrial and glycolytic function

**Fig. 3:**
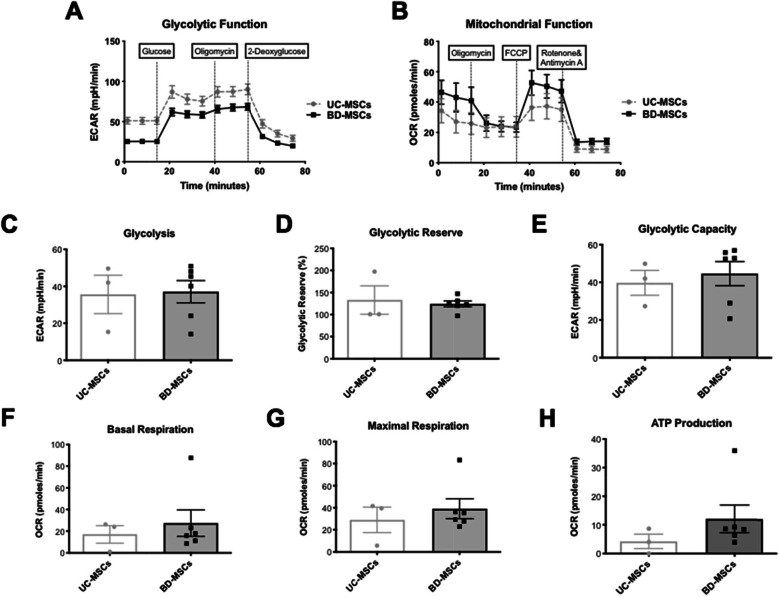
Mitochondrial and glycolytic function. **a**, **c**–**e** Glycolytic function, ECAR = extracellular acidification rate. Glycolysis. Glycolytic Reserve. Glycolytic Capacity. **b**, **f**–**h** Mitochondrial function, OCR = oxygen consumption rate. Basal Respiration. Maximal Respiration. ATP Production. Statistical significance is indicated with asterisks: *p value ≤0.05, ** *p* ≤ 0.01, and *** *p* ≤ 0.001. No asterisks represent *p* > 0.05. *N* = 3 for each group (=cells from 3 different patients per group); 6 replicates per biological sample. Graph: A and B with SEM. **c**–**h** Mean with SEM

No statistical difference could be found between BD-MSCs and UC-MSCs in terms of their mitochondrial function. However, BD-MSCs showed a signal toward higher oxidative metabolism due to their slightly higher basal respiration (basal respiration 27.3 ± 12 pmol/min and 17.1 ± 8 pmol/min; maximal respiration 39.1 ± 9 pmol/min and 28.9 ± 12 pmol/min; adenosine triphosphate (ATP) production 18.7 ± 17 pmol/min and 4.2 ± 3 pmol/min in UC-MSCs) (Fig. 3b, f–h).

In addition, there was no statistical difference between BD-MSCs and UC-MSCs in terms of their glycolytic function (glycolysis 37.1 ± 6mpH/min and 35.7 ± 1mpH/min, respectively; glycolytic reserve 124.2 ± 6mpH/min and 132.9 ± 32mpH/min; glycolytic capacity 44.6 ± 6mpH/min and 39.7 ± 7mpH/min) (Fig. 3a, c–e).

### Immunologic surface markers

To potentially predict immunologic rejection and inflammation after cell grafting, the expression of major histocompatibility complex (MHC) I and II molecules as well as of toll-like receptors (TLR) were assessed.

**Fig. 4:**
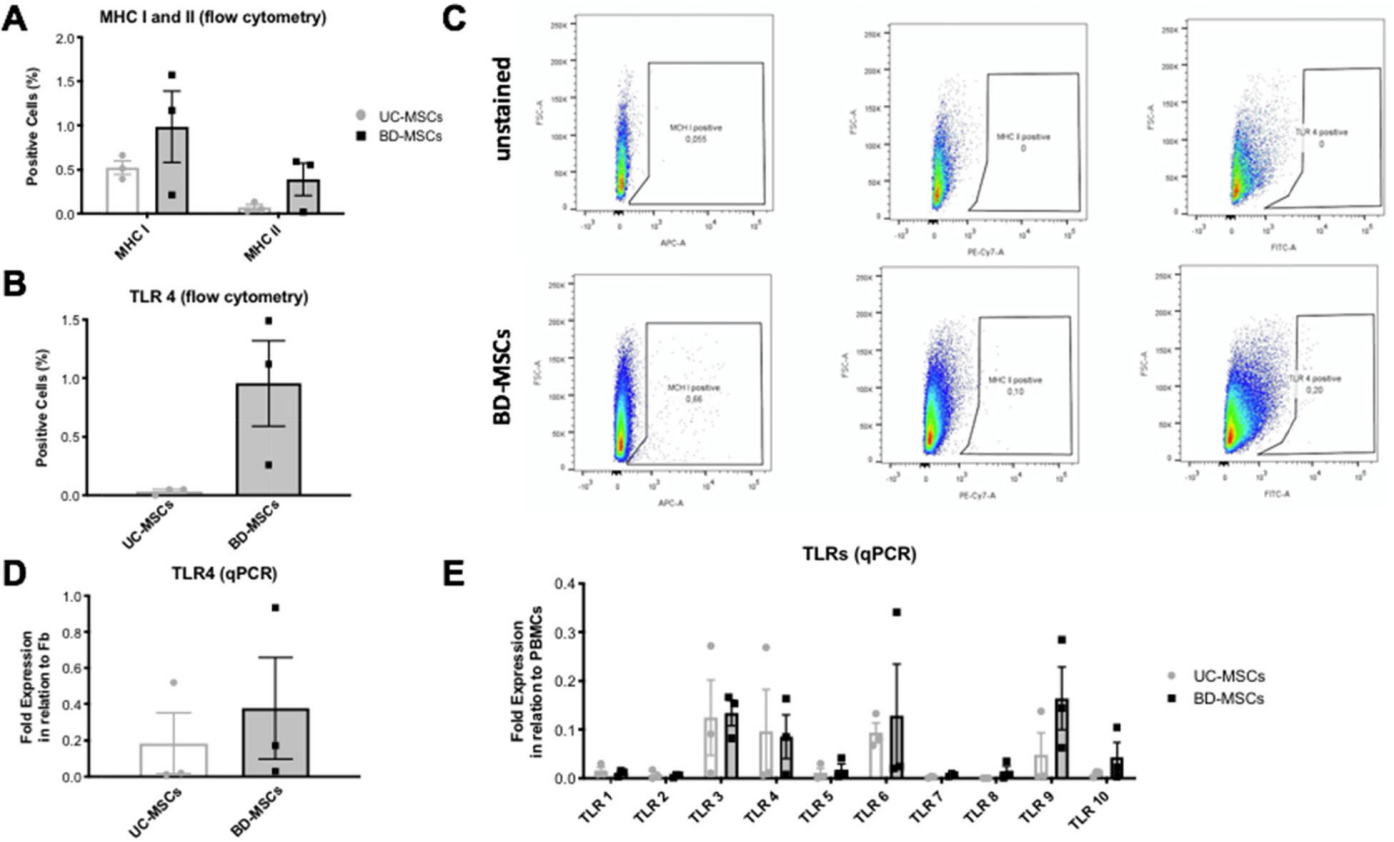
Immunologic surface markers. **a** MHC complex I and II expression. **b** Toll-like receptor (TLR) 4 expression. **c** Flow cytometry of BD-MSCs (passage 3) stained for MHC I, II, and TLR 4. The upper panel shows unstained cells. **d**, **e** Quantitative PCR (qPCR) expression of TLR 4 compared to human fibroblasts (Fb). **e** QPCR expression of TLR 1–10 expression compared to human peripheral blood mononuclear cells without statistical significance. Statistical significance is indicated with asterisks: *p value ≤0.05, ** *p* ≤ 0.01, and *** *p* ≤ 0.001. No asterisks represent *p* > 0.05. *N* = 3 for each group (=cells from 3 different patients per group); triplicates per biological sample. Graph: Mean with SEM

Both, BD-MSCs and UC-MSCs showed a very low expression of MHC I and II in flow cytometry readings, without any statistically significant difference (MHC I: 0.98 ± 0.04% and 0.37 ± 0.01%, respectively; MHC II: 0.52 ± 0.09% and 0.07 ± 0.6%) (Fig. 4a, c).

No statistical difference in the expression of TLR 1–10 could be found between BD-MSCs and UC-MSCs in qPCR (Fig. 4e). However, BD-MSCs showed a signal toward higher expression of TLR-4 in qPCR (Fig. 4d) which could be confirmed in flow cytometry (1 ± 0.03% of BD-MSCs pos. for TLR-4 vs. 0.01 ± 0.01% of UC-MSCs) (Fig. 4b).

### Cytokine, chemokine, and growth factor expression

The expression of cytokines, chemokines, and growth factors was measured at baseline and after stimulation with bacterial lipopolysaccharide (LPS).

**Fig. 5:**
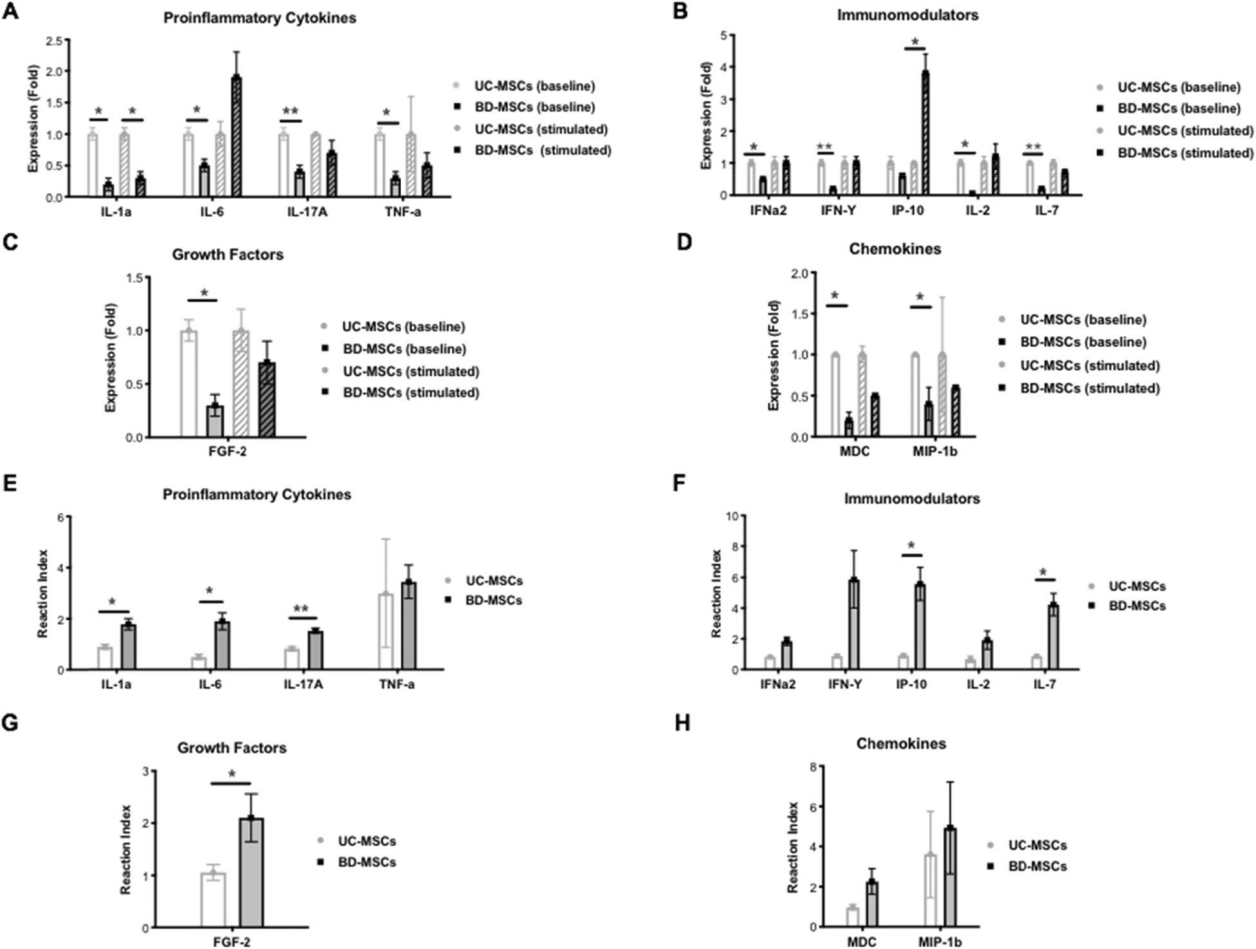
Cytokine, chemokine, and growth factor expression. **a**–**d** Assessment of cytokine, chemokine, and growth factor expression, before and after stimulation with bacterial LPS on UC- and BD-MSCs. **a** Proinflammatory Cytokines. **b** Immunomodulators. **c** Growth Factors. **d** Chemokines. **e**–**h** Assessment of cytokine, chemokine, and growth factor expression, shown as the reaction index after LPS stimulation. Statistical significance is indicated with asterisks: *p value ≤ 0.05, ** *p* ≤ 0.01, and *** *p* ≤ 0.001. No asterisks represent *p* > 0.05. *N* = 3 for each group (=cells from 3 different patients per group); triplicates per biological sample. Graph: Mean

When compared with UC-MSCs, BD-MSCs showed a lower baseline expression of all 34 assessed cytokines, chemokines, and growth factors with 11 reaching statistically significance. Proinflammatory cytokines are IL-1a (*p* = 0.02), IL-6 (*p* = 0.03), IL-17a (*p* = 0.003), and TNFa (*p* = 0.01). Immunomodulatory cytokines are IFNa2 (*p* = 0.047), IFN-Y (*p* = 0.004), IL-2 (*p* = 0.01) and IL-7 (*p* = 0.004). Growth factor is FGF-2 (*p* = 0.01). Chemokines are MDC (*p* = 0.01) and MIP-1b (*p* = 0.04) (Fig. 5a–d).

After LPS stimulation, only IL-1a continued to be significantly more secreted by UC-MSCs (*p* = 0.01) compared to BD-MSCs (Fig. 5a). IP-10, which showed no difference in the baseline secretion, was significantly higher expressed in BD-MSCs after LPS stimulation compared to UC-MSCs (*p* = 0.04) (Fig. 5b).

BD-MSCs displayed a higher reactivity to LPS stimulation in all 34 parameters compared to UC-MSCs with 7 reaching statistical significance: IL-1a (*p* = 0.04), IL-6 (*p* = 0.02), IL-17a (*p* = 0.007), IP-10 (*p* = 0.047), IL-7 (*p* = 0.04), IL-8 (*p* = 0.01), and FGF-2 (*p* = 0.046) (Fig. 5e–h).

## Discussion

Here we demonstrated that BD-MSCs extracted from full-thickness burns are functional MSCs with equal overall similarities to UC-MSCs. Furthermore, BD-MSCs seem to be unaffected by the thermal damage in regard to key cell functions and are comparable to mesenchymal stem cells extracted from Wharton’s jelly of human umbilical cords (UC-MSCs). This is an important finding for regenerative medicine and the wound healing community aiming to regenerate skin.

The BD-MSCs differentiated to all three mesenchymal lineages. However, BD-MSCs did show a lower mesenchymal differentiation capacity compared to UC-MSCs for adipogenic and chondrogenic differentiation in the in vitro experiment. This could be due to the higher multipotent differentiation potential of the young UC-MSCs, which are faster in cell differentiation compared to the adult MSCs [6, 34]. Another explanation could be that the extracted cells of the burn tissue contain a subpopulation [36] of very differentiated progenitor-MSC-like-fibroblasts [3] of the mesenchymal lineage. These lineage shares the same three positive surface markers as per definition of MSCs [37].

In the evaluation of basic cell functions, such as population doubling time, colony formation, cell proliferation/cell cycle, and ROS release, we found both cell types were similar. In general, MSCs have a great ability to reduce ROS [28, 38], which is beneficial for regeneration. Interestingly though, skin-MSCs, have been described to reduce less ROS. For instance, fibroblasts exposed at 43 °C for 30 min showed an increase in damage from oxidative stress [39], which preceded cellular apoptosis [40]. Skin-MSCs die may be due to the fact that the basal membrane [41] and dermis [42] are highly dynamic and continuously regenerating and exfoliating. Therefore, the progenitor skin cells are not interested in investing energy in cell repair, as shown in the very outer layer as keratinocytes [37, 43], and the mechanism in reducing ROS as the precursor cells. However, the literature also indicates that a longer exposure in these mentioned examples for 30 min [41] to 2 h [40] is necessary to create cell damage. We do not know how long our samples were exposed to the burn; however, a scalding exposure usually occurs within seconds. This avenue needs to be further explored due to the complexity of cell damage [44] and to be able to make adequate comparisons [45] in BD-MSCs.

In our Seahorse experiments, we assessed cell metabolism by analyzing glycolytic and mitochondrial functions to determine if the thermal injury altered these metabolic components. The BD-MSCs did not differ from UC-MSCs, but a signal toward higher oxidative phosphorylation was found. This is not surprising since it was shown that heat increases mitochondrial respiration [41]. However, the main energy-gaining mechanism in MSCs, the anaerobic glycolysis that is responsible for the majority of the ATP production [46, 47], was similar and not significantly different, even though a single outlier was seen in the ATP production, the basal as well as maximal respiration. Overall, these bioenergetic cellular results suggest that BD-MSCs maintained their ability to produce rapid energy with anaerobic glycolysis.

For considerations regarding transplantation (i.e., graft-versus-host disease, GvHD), the immunosuppressive properties were assessed [48]. We found both MSCs had low expression of the MHC complexes I and II, which is in conjunction with previous findings, especially described in the immune privileged UC tissue [49], which suggests low immunogenicity and immunoreactivity potential of the evaluated cells, and further shows that the burn exposure does not affect the safety for future cell grafting. Therefore, the predicted interaction in the case of transplantation should be low.

The toll-like receptors (TLR), which are found in humans (TLR 1–10) [23] and are involved in T cell-receptor mediated interactions, are expressed by various immune (i.e., peripheral blood mononuclear cells, PBMC) and non-immune cells (i.e., fibroblasts). Specifically, TLR4 has been assessed due to its dual role post-burn [50]. First, burn wounds, like every other wound, are heavily colonized by bacteria (within < 48 h), especially *Pseudomonas aeruginosa*, a gram-negative bacteria which is problematic in burn wounds [51, 52] and associated with the highest lethality [53, 54] from sepsis [49]. Second, prolonged inflammation leads to fibrotic healing [7], and in the bigger picture, to scarring and skin contracture formation, which leads to a significant personal and socioeconomic burden [7, 55] as well as aesthetical stigma for patients. Therefore, it is of great interest to determine possible cell surface receptor interactions for future investigations to understand to what extent the applied cells react [55–57] to various bacterial, fungal, and viral/infectious stimuli. We found neither a difference nor a significant increase in any TLRs in our comparison of BD- and UC-MSCs. TLR-4 showed a higher signal, which could be confirmed in a panel of a qPCR test, where elevated levels were similarly found in TRL 3, 4, 6, and 9, as well as 10 in BD-MSCs. The exact purpose of all these TLR is unknown. However, it is known that the activation of TLR 3 and 4 on MSCs leading to recruitment and promotion of immunosuppressive regulatory T cells [46, 58]. In addition, TLR also seems to play a role in cell proliferation and differentiation of MSCs toward progenitors, as shown in an example in osteogenesis [59]. Taken together, BD- and UC-MSCs with similarly elevated levels of cytokines, chemokines, and growth factors might boost beneficial inflammatory recruitment, reduce wound inflammation, and enhance bacterial clearance [60]. Further in vivo analysis needs to be conducted.

We investigated the essential cytokines, chemokines, and growth factors from both BD-MSCs and UC-MSCs by measuring their levels. Of the 34 assessed proteins, BD-MSCs only showed a significantly different expression of 11 at baseline and 2 at stimulated LPS conditions simulating a bacterial, fungal, and viral infectious environment. BD-MSCs, like skin cells, seem to be slightly more reactive to LPS than UC-MSCs. Assessing the absolute expression output, the UC-MSCs seem to be the more potent cells based on the classic example of the growth factor FGF-2 production as previously described [15]. As other researchers described previously, we observed a similar cytokine expression pattern, such as IP-10 [61, 62] and increased IL-6, IL-17, and TNF-alpha for reparative cells [63], in secretory cytokine profiling from both MSCs. MSC-secreted cytokines are involved in proinflammatory reactions, cell differentiation, activation, and proliferation of leukocytes (i.e., macrophages), endothelial cells, keratinocytes, and fibroblasts.

The data suggest that BD-MSCs have comparable reactions and overall do not significantly differ in the expression profile of cytokines, chemokines, growth factors, and immunomodulatory proteins when compared to UC-MSCs. MSCs, known for their ability to evade immunologic rejection, can induce beneficial immunomodulatory effects [5]. Previously, our group translated the presented results in a first efficacy and safety study, where we successfully grafted BD-MSCs on murine and pig models, without creating tumorgenicity, and found enhanced wound healing using these cells compared to a standard acellular control used in the clinic for burn treatment, rendering BD-MSCs a promising candidate for skin regeneration [6].

### Limitations

A limitation of this study lies in the small sample size of *N* = 3 for BD-MSCs and UC-MSCs, with 6 replicates per biological sample, respectively. This sample size, however, is not uncommon in stem cell research due to limited cell availabilities and high costs [64–67]. Even though the sample size does not allow for the conclusive evidence that BD-MSCs are equal as UC-MSCs, it highlights its general potential as a source for skin regeneration. Many research questions remain unknown; this study opens the discussion for new avenues throughout the stem cell and burn community. We only used skin from burns via scalding to avoid confounding. It is unclear whether BD-MSCs extracted from electrical, chemical, or flame burns have the same properties as BD-MSCs described here. Several factors like aging and the health status of the donor may affect the extent of extracted cells as well as their functionality. Having a larger sample size could minimize the effect of these confounding factors in an investigation targeting specific research questions.

### Future directions

Future investigations in multiple directions as, for instance, their heat tolerance capacity [68] after a burn trauma and their exact origin, are warranted to further understand those cells in detail, which will be of great academic importance. The crucial question is why cells survive a burn and if such injuries activate further down-stream mechanism, including crucial survival and repair mechanism and ultimately initiation of epigenetic factors. Answers to these questions will contribute in the discussion if burned-derived cells are unique and could be a potential source in burn care, and help in the current discussion of the ethical issue of potential over-debridement in burn surgery.

## Conclusion

The recent discovery of BD-MSCs offers a potential new source for autologous cell transplants in burn patients. While our previous data showed the functionality of these cells in vivo, little was known about the cellular characteristics. This study is the first to show that these cells do not show impairment in more key biological functioning, compared to MSCs extracted from Wharton’s jelly of the human umbilical cord, despite the thermal injury that had required the excision of the skin. The expression of immunological surface markers (TLR and MHC I and II), responsible for immunologic rejection and inflammatory responses, is low and comparable to MSCs from umbilical cords that are already successfully used for cellular therapy. This study contributes to a better understanding of BD-MSCs and their potential role as a cellular graft in burn patients. Further trials are warranted to fully evaluate potential heat-related alterations as well as their role in tissue regeneration.

### Wording and definition

Stem cells have functional properties as per definition. For multipotent mesenchymal stromal cells (MSCs), we refer to the in vitro definition of characterization from the International Society for Cellular Therapy (ISCT; Position Statement, 2006). Clonogenicity in vitro is used to define the stromal progenitors. As upstream precursors, we define any cells/subsets derived from the skin sharing the three positive cell surface markers as MSCs.

## Supplementary Information

**Additional file 1.**

## Data Availability

The datasets generated and analyzed during the current study are available from the corresponding author on reasonable request. This manuscript was written after publishing 2017 the Master thesis of the first author (and is available on: http://hdl.handle.net/1807/79267).

## Abbreviations

ATP: Adenosine triphosphate
BD-MSC: Burn-derived mesenchymal stem cell
BrdU: Bromodeoxyuridine
CD: Cluster of differentiation
FBS: Fetal bovine serum
LPS: Lipopolysaccharide
MHC: Major histocompatibility complex
MSC: Mesenchymal stem cells, multipotent stromal cell
ROS: Reactive oxygen species
PDT: Population doubling time
TLR: Toll-like receptor
UC-MSC: Umbilical cord mesenchymal stem cell
