# Supplementary material for: Biological characteristics of stem cells derived from burned skin—a comparative study with umbilical cord stem cells
Source: Stem Cell Res Ther. 2021 Feb 17;12:137. doi: 10.1186/s13287-021-02140-z (PMC7888080; doi:10.1186/s13287-021-02140-z)
Supplement: Supplementary file 1 — Additional file 1. [file 13287_2021_2140_MOESM1_ESM.docx]

**SUPPLEMENTARY MATERIAL**


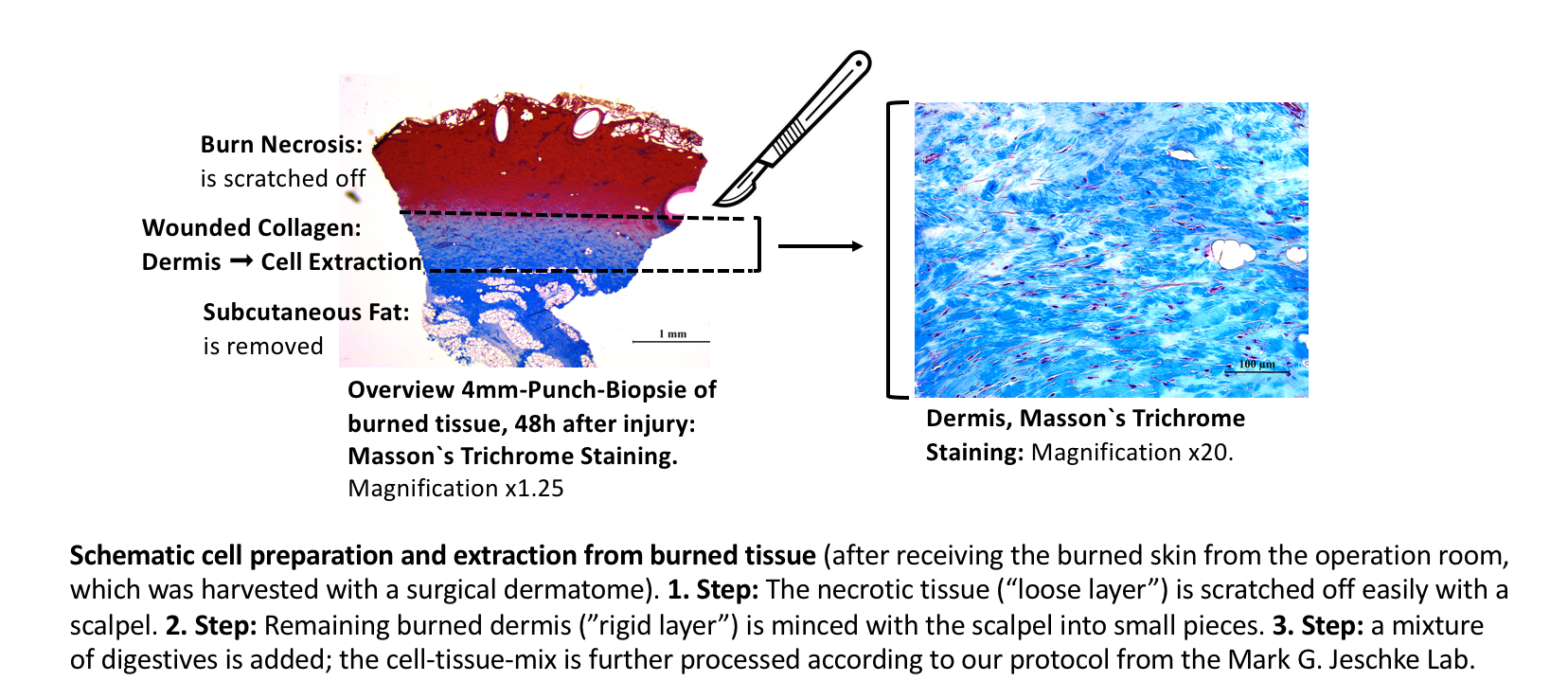


© Gertraud Eylert, 2020.

Protocol:

1. Burned skin was received (without subcutaneous fat; harvested with a surgical dermatome) from the surgeon and cut into small pieces. The tissue was washed for 30 sec in 70% ethanol in a 50ml tube, followed by 30sec in Phosphate Buffered Saline 1x (PBS, Multicell, Wisent Inc., USA) containing 1% Ab/Am (Gibco Antibiotic-Antimycotic, Thermo Fischer Scientific, Canada), and by 30sec in PBS containing 2% Ab/Am.
2. The necrotic part was removed (scratched off), the dermal part was cut and minced into small pieces, and homogenized with a sterile scalpel until no macroscopical pieces could be identified anymore.
3. The tissue homogenate as diluted with an enzyme solution (1:2) in a 50ml tube. The enzyme solution was pre-mixed in 50ml quantities:
4. 135mg of collagenase 1 (Worthington Biochemical Corporation, USA)
5. 2.5ml dispase (Life Technologies Corporation, USA)
6. 10ml 0.05% trypsin (Life Technologies Corporation, USA)
7. 37.5ml Dulbecco`s Modified Eagle Medium (Gibco DMEM, Thermo Fischer Scientific, USA) with 2% antibiotic-antimycotic solution (Gibco Antibiotic-Antimycotic, Thermo Fischer Scientific, Canada).
8. The 50ml tube containing the homogenate and the enzyme mix, was incubated at 37 degrees Celsius under constant rotation for 60-70minutes.
9. The viscous cell/enzyme mix-solution was diluted with PBS (1:5) and filtered through a sterile 10μm cell strainer (Falcon® 10μm Cell Strainer, Corning, USA) into a new 50ml tube.
10. After dilution and filtering, the tubes were spun down at 1600rpm for 10min.
11. After supernatant was removed via vacuum suctioning.
12. The remaining cell pallet was diluted with 7-12ml Dulbecco`s Modified Eagle Medium enriched with 1% Ab/Am and 10% fetal bovine serum (FBS, Gibco fetal bovine serum, Thermo Fischer Scientific, Canada).
13. The 7-12ml cell/media mixture was given into either 75cm2 cell culture flasks or 6cm standard cell culture dishes.

**Supplementary Material Figure 1. Cell preparation and extraction protocol.**
